# Mobile phone addiction and depressive symptoms among Chinese University students: The mediating role of sleep disturbances and the moderating role of gender

**DOI:** 10.3389/fpubh.2022.965135

**Published:** 2022-09-20

**Authors:** Meng Liu, Chuntian Lu

**Affiliations:** Department of Sociology, School of Humanities and Social Sciences, Xi'an Jiaotong University, Xi'an, China

**Keywords:** mobile phone addiction, depressive symptoms, sleep disturbances, University student, gender

## Abstract

**Background:**

With the continuous updating of mobile phone functions, the phenomenon of mobile phone addiction among University students is becoming more and more serious. It is important to identify the potential risk factors for mobile phone addiction. The aim of the study was to examine whether there is a relationship between mobile phone addiction and depression symptoms in University students, and to investigate whether sleep disturbances play a mediating role between mobile phone addiction and depression symptoms, as well as the moderating role of gender.

**Methods:**

A cross-sectional study, carried out between September to December 2021, recruited 973 students (478 males) from seven comprehensive universities in western China. The Mobile Phone Addiction Index (MPAI), the Patient Health Questionnaire-9 (PHQ9), and the Pittsburgh Sleep Quality Index (PSQI) were used to complete measures of mobile phone addiction, depressive symptoms, and sleep disturbances. For statistical analyses, descriptive statistics, correlation, regression, mediation and moderated mediation analyses were used. Furthermore, we tested the mediation model and moderated mediation model using the SPSS macro PROCESS.

**Results:**

In this study, it was found that there were positive correlations between mobile phone addiction and depressive symptoms among Chinese University students. Mediation analyses revealed that this relationship was partially mediated by sleep disturbances, but the mediating role was not moderated by gender.

**Conclusion:**

Sleep disturbances have a partial mediating role in the relationship between mobile phone addiction and depressive symptoms. Our results highlight the critical role of prevention and early identification of mobile phone addiction among University students, especially those with sleep disturbances.

## Introduction

As an excellent carrier of mobile Internet development, mobile phones have opened up new interactions and experiences in the fields of social communication, access to information, m-commerce, m-learning, and the healthcare system (1–4). However, the versatility of mobile phones has led to an increasing number of people overusing them, especially among University students (5). Overuse of mobile phones is now often associated with potentially harmful and/or destructive behaviors, and this problematic mobile phone use has generally been conceptualized as a behavioral addiction (6, 7). Mobile phone addiction can be regarded as an impulse control disorder, which refers to behavioral and emotional problems caused by uncontrolled, inappropriate or excessive use of mobile phones, and a compulsive state in which an individual's physiological, psychological, and/or social functions are impaired (8–10).

Mobile phone addiction could lead to negative health outcomes. Studies have indirectly shown that there may be biological and psychological links among mobile phone addiction, sleep disturbances, and depressive symptoms. Mobile phone addiction is a risk factor for sleep disturbances, prolonged mobile phones use is significantly linked to insomnia and shortened sleep duration (11–14). Moreover, constant exposure to blue light suppresses melatonin production, causing circadian disruption, sleep disturbances, and mental overload, which might be an important factor in developing psychopathological symptoms, such as depression (15, 16). Mobile phone addicts are more likely to adopt unhealthy lifestyles, which could also be considered as predisposing factors for depression (17). Therefore, mobile phone addiction could lead to serious psychological problems such as depression symptoms, anxiety, and serious health problems, such as sleep disturbances (18, 19).

Despite the extensive research on the relationship between mobile phone addiction, depression symptoms, and sleep disturbances, previous findings are inconsistent or comprehensive, and more research is needed to confirm these findings in different social and cultural contexts. To fill these research gaps, we conducted the study in a sample of Chinese University students. In the present study, we aimed to determine whether prevention and reduction of mobile phone addiction could help alleviate depressive symptoms among Chinese University students. To further understand whether sleep disturbances can be improved by reducing mobile phone addiction, and depressive symptoms could be alleviated by improving sleep disturbances.

## Literature review and research hypotheses

In recent years, studies have reported the association between mobile phone addiction and depression among University students in different countries. Specifically, a series of cross-sectional research revealed that mobile phone addiction is positively associated with depressive symptoms among college students in Turkey, Japan, South Korea, Austrian, and Lebanon (20–25). A prospective cohort study found that a bidirectional longitudinal relationship between duration of mobile phone use and severity of depression (26). A meta-analysis of 40 studies provided solid evidence that mobile phone addiction was positively correlated with depressive symptoms and sleep disturbances among University students (27). A study using a sample of 188 University students in South Korea found that there was a significant relationship exists between mobile phone addiction and depressive symptoms, but there was no strong correlation between sleep disturbances and mobile phone addiction (23). However, a study conducted among students at Surat University, India showed that there was a moderate correlation between mobile phone addiction and depression, as well as between mobile phone addiction and sleep quality (28). Apart from this, conclusions from a systematic review and meta-analysis of included studies published between 2010 and 2019 showed that mobile phone addiction was significantly associated with increased risk of sleep disturbances and depressive symptoms among University students, and that sleep quality was influenced by multiple factors, including gender (29). The effects of mobile phone addiction on one's emotions are likely to be mediated by other variables, rather than a direct effect (27). Therefore, the association between mobile phone addiction and depression symptoms needs to be examined in more diverse samples to further explore the underlying mechanism involved in the process. The study demonstrated that problematic mobile phone use was associated with mental health among University students, and sleep quality played a mediating role in this relationship (30). However, a study conducted among Turkish University students reported that depressive symptoms as a mediator between mobile phone overuse and sleep disturbances (20). The following subsections detail the specific arguments regarding these relationships in this study and present the underlying rationale.

### Mobile phone addiction and depressive symptoms

Depression, as a negative emotion, refers to a cluster of repetitive and significant internalized problems, with low mood, poor initiative, low self-esteem, feelings of uselessness loss of interest in activities once enjoyed, etc., that have persisted for some time and caused distress to the individual (31, 32). The concept of reciprocal determinism in Bandura's (1986) social cognition theory (33) points out that there is an interplay between individuals' behavior and emotion is reciprocal, and individuals' behavior not only reacts to emotion but also affects their emotion (34). That is to say, individuals' use of mobile phones may produce depressive symptoms, while individual depression caused by excessive use of mobile phones may also have an impact on individual mood. At present, there are multiple literatures discussing the negative emotions represented by depression leading to problematic mobile phone use (35, 36). Compensatory Internet use theory, for example, suggests that people with depressive symptoms may tend to use the Internet to avoid current negative feelings, which increases motivation to go online (37). As a portable surfing tool, mobile phones are convenient for people to surf the Internet, which may aggravate problematic mobile phone use and develop into mobile phone addiction. Therefore, our study focuses on the first half of the pathway of the concept of reciprocal determinism.

In recent years, empirical evidence linking mobile phone addiction levels to depressive symptoms has rapidly accumulated. Although studies have been carried out among University students in China, the relationship between mobile phone addiction and depressive symptoms has rarely been examined in a sample of students from comprehensive universities in western China. Therefore, University students in western China were selected as samples for this study. We put forward the following hypothesis:

Hypothesis 1: Mobile phone addiction would positively predict depressive symptoms among Chinese University students.

### The mediating role of sleep disturbances

One of the central factors of mobile phone addiction was personal dependence, and its consequences include stress, mental overload, role conflicts, sleep disturbances, feelings of guilt due to inability to achieve set goals, and so on, which are major sources of depressive symptoms (16). Brand et al. (38) proposed an updated version of the Interaction of Person-Affect-Cognition-Execution (I-PACE) model. This model is a theoretical approach to describe the process of addictive behaviors by combining the individual psychological and neuroscientific theories of substance use disorder and behavioral addiction. It explains a series of addictive behaviors, including mobile phone addiction. The model proposes that the disorders caused by addictive behaviors are the consequence of the interaction between a person's core characteristics and several moderating and mediating variables. The model also points out that these variables may not be static but dynamic, developing over time as a result of engaging in specific behaviors. In this study, we identified sleep disturbances as a disorder due to mobile phone addictive behaviors. Here, the second hypothesis was raised:

Hypothesis 2: Sleep disturbances would play a mediating role between mobile phone addiction and depressive symptoms.

Hypothesis 2a: Mobile phone addiction would positively predict sleep disturbances among Chinese University students.

Hypothesis 2b: Sleep disturbances would positively predict depressive symptoms among Chinese University students.

### The moderating role of gender

According to our hypothesis, mobile phone addiction may predict depressive symptoms through the effect of sleep disturbances, while the intensity of this mediation effect is unlikely to be identical for all the University students. Although a theme of interest to many researchers is related to gender differences in mobile phone addiction, the role of gender as an individual characteristic in the formation of mobile phone addiction is far from clear. Previous studies have shown that there were differences between male and female in sleep quality and mobile phone use preference among young adults. Females have better sleep quality than males, they had shorter sleep onset latency, better sleep efficiency (39). Males were more likely to play online games, watch mobile videos and listen to music, while females were more likely to use social networking services and mobile phone communication functions (40). In other words, females answer phone calls and text messages more often. While young adult females reported better sleep quality, spending an amount of time answering phone calls and texts after sleep onset could be an emotional component or sleep hygiene issues that lead to sleep disturbances (41).

Studies demonstrated that mobile phone addiction was associated with sleep disturbances for the male (42). However, other studies have found that the moderating effect of gender on mobile phone use is inconclusive (43). Based on the I-PACE model, we argue that gender may play a role in this process. To be specific, this study will test whether the association between mobile phone addiction and sleep disturbances in University students would be moderated by their gender. Here we put forward the third hypothesis:

Hypothesis 3: Gender would moderate the relationship between sleep disturbances and depressive symptoms. Specifically, mobile phone addiction would result in more depressive symptoms for female University students.

## The present study

Based on the literature review mentioned above, the present study constructed a moderating effect model to examine the mediating role of sleep disturbances between mobile phone addiction and depressive symptoms among Chinese University students. In addition, we tested whether the indirect path between mobile phone addiction and sleep disturbances would be moderated by their gender.

## Methods

### Study design and participants

Multi-stage stratified random sampling method was conducted to randomly select students from seven comprehensive universities, which are Shaanxi Normal University, Tianshui Normal College, Xi'an University of Electronic Science and Technology, Xi'an University of Technology, Xi'an Jiaotong University, Xi'an University of Technology, and Xi'an University of Science and Technology (including many disciplines and majors, such as humanities and social sciences, medicine, science and technology) in western China for questionnaire survey. Our questionnaire was issued from September 13 to December 12 in 2021. Firstly, we randomly selected different classes from each University. After that, paper questionnaires were distributed to each participant, who was guided by well-trained investigators to complete the written questionnaire within 20 min, so as to ensure the validity of the questionnaire. It should be noted that the investigators' guidance has not been involved the discussion of the questionnaire contents, but only for the filling in the instructions, such as explaining the reserved time and indicating where to fill in the answers. The inclusion criteria were full-time students without jobs. One Thousand and forty-four students were recruited. Of all participants, 71 students had to be excluded for missing or unreliable data on some vital study variables. After excluding the invalid data, the valid sample size was 973 University students (478 of them were male).

### Procedure

The study protocol was approved by the Biomedical Ethics Committee of Xi'an Jiaotong University. Verbal informed consent was obtained from participants before the data collection. Participants were told that their participation was entirely voluntary and that they could terminate at any time. They were also told that all the data collected were anonymous and that their responses would be kept confidential. Written informed consent to participation was not required for this study in accordance with the national legislation and the institutional requirements.

## Measures

### Sociodemographic characteristics

Demographic data of participants included gender, age, residence (1 = rural; 2 = urban), and self-rated health. Participants ranged in age from 17 to 25 years old (M = 19 years old, SD = 1.04). More than half of the participants came from urban areas (55.9%). Most of participants' self-rated as being in good health (44.3%). In the full sample, the mean score of the Mobile Phone Addiction Index (MPAI) was 36.03 (SD = 12.66). The mean score of the Patient Health Questionnaire-9 (PHQ9) was 4.34 (SD = 4.15), and the mean score of the Pittsburgh Sleep Quality Index (PSQI) was 5.46 (SD = 2.71). The role of gender was one of the main concerns in our study, the descriptive information about variables in the male and female groups showed that the average scores of females were higher than that of males in the three scales. The current sample reported moderate sleep disturbances. Among the seven dimensions of sleep disturbances, except for the two dimensions of sleep duration and habitual sleep efficiency, which reported slightly higher scores for males, while the other five dimensions reported higher average scores for females (see Table 1).

**Table 1:** Participant characteristics for the entire sample and by gender (*n* = 973).

**Variables**	**Full sample**			**Male (*****N*** = **478)**		**Female (*****N*** = **495)**		***P*-value**
	***N* (%)**	**Mean (SD)**	**Range**	***N* (%)**	**Mean (SD)**	***N* (%)**	**Mean (SD)**	
Age	973 (100.0)	19 (1.04)	17-25	478 (100.0)	19.15 (1.70)	495 (100.0)	19.47 (1.92)	0.003
Residence	970 (99.7)			476 (99.6)	1.44 (0.50)	494(99.8)	1.44 (0.50)	0.995
Rural	544 (55.9)			267 (55.9)		277(56.0)		
Urban	426 (43.8)			209 (43.7)		217 (43.9)		
Self-rated health	973 (100.0)	2.36 (0.81)		478 (100.0)	2.31 (0.87)	495 (100.0)	2.41(0.75)	0.59
In excellent health (1)	133 (13.7)			85 (17.8)		48 (9.7)		
In good health (2)	431 (44.3)			201(42.1)		230 (46.4)		
General health condition (3)	341 (35.0)			156 (32.6)		185 (37.3)		
In poor health (4)	64 (6.6)			32 (6.7)		32 (6.5)		
In very poor health (5)	4 (0.4)			4 (0.8)		0 (0.0)		
MPAI	930 (95.6)	36.03 (12.66)	17–80	455 (95.2)	34.06 (12.04)	475 (96.0)	37.91 (12.96)	<0.001
PHQ9	960 (98.7)	4.34 (4.15)	0–27	473(99.0)	4.00 (4.39)	487 (98.4)	4.67(3.88)	0.13
PSQI	921 (94.7)	5.46 (2.71)	0–17	450 (94.1)	5.12 (2.70)	471 (95.2)	5.79(2.68)	<0.001
Subjective sleep quality	966 (99.3)	0.95 (0.67)	0–3	473 (99.0)	0.93 (0.69)	493 (99.6)	0.98 (0.66)	0.27
Sleep latency	958 (98.5)	0.97 (0.81)	0–3	470 (98.3)	0.91 (0.80)	488 (98.6)	1.03 (0.81)	0.24
Sleep duration	965 (99.2)	1.10 (0.73)	0–3	474 (99.2)	1.10 (0.72)	491(99.2)	1.10 (0.73)	0.99
Habitual sleep efficiency	962 (98.9)	0.19 (0.52)	0–3	471 (98.5)	0.20 (0.53)	491 (99.2)	0.19 (0.51)	0.67
Sleep disturbance	941 (96.7)	0.86 (0.54)	0–3	462 (96.7)	0.78 (0.54)	479 (96.8)	0.93 (0.53)	<0.001
Use of sleep medication	968 (99.5)	0.06 (0.38)	0–3	476 (99.6)	0.05 (0.35)	492 (99.4)	0.08 (0.40)	0.20
Daytime dysfunction	968 (99.5)	1.35 (1.00)	0-3	475 (99.4)	1.16 (1.00)	493 (99.6)	1.53 (0.97)	<0.001

Analysis between male vs. female was performed with *t*-tests for continuous variables (normal distribution) and χ^2^ test for categorical variables.

SD, Standard Deviation; MPAI, Mobile Phone Addiction Index; PHQ9, Patient Health Questionnaire- 9; PSQI, Pittsburgh Sleep Quality Index.

### Mobile phone addiction

Mobile phone addiction was assessed using the Chinese version of the Mobile Phone Addiction Index (MPAI) (9), which was compiled based on the twenty-seven-item Mobile Phone Problem Use Scale (MPPUS) (44). This is a self-report questionnaire that involves rating 17 items and representative items were “Your friends and family have complained about you using your mobile phone.”, “You try to spend less time on your phone, but you can't.”, “You find yourself addicted to mobile phone when there are other things you need to do, and that can cause you some trouble.”. The four factorial components of the scale were inability to control craving, feeling anxious and lost, withdrawal or escape, and productivity loss. The measurement was based on a Likert 5-point scale ranges from “1 = Not at all” to “5 = Always”, with higher scores indicating higher levels of mobile phone addiction. For the current study, Cronbach's alpha for this scale was 0.913, demonstrating good internal consistency.

### Depression symptoms (PHQ-9)

Patient Health Questionnaire-9 (PHQ-9) (45) is a 9-item self-reported questionnaire that assesses the major symptoms of depression outlined in Diagnostic and Statistics Manual of Mental Disorders- Fourth Edition (DSM-IV) (46) (e.g., “Having little interest or pleasure in doing things.”, “Feeling tired or having little energy.”, and “Loss of appetite or overeating.”), the items are scored on a 4-point Likert scale (0 = Not at all, 1 = Several days, 2 = More than half of the days, and 3 = Nearly every day). Participants rated the frequency of depressive symptoms over the past 2 weeks (47). The PHQ-9 had been translated into Chinese, and the Chinese version of the PHQ-9 has been shown to be a valid and efficient tool for screening depression (48). The total score of the nine items ranges from 0 to 27, indicating severity of depression symptoms (0 indicating no depression symptoms and 27 indicating that all symptoms occur nearly daily). The Cronbach's alpha of the scale was 0.868, indicating good internal consistency of the measure.

### Sleep disturbances (PSQI)

The Chinese version of the Pittsburgh Sleep Quality Index (PSQI) (49) was used in the study and its reliability was satisfactory (Cronbach's alpha = 0.81). The index is a self-administered questionnaire with 19 individual items which assesses sleep quality and sleep disturbances during a 1-month period. The 19 items are divided into seven components, including a participant's subjective sleep quality, sleep latency, sleep duration, habitual sleep efficiency, sleep disturbance, use of sleep medication, and daytime dysfunction. A Likert 4-level scoring method was used for each dimension (0 = Not at all, 1 ≤ Once a week, 2 ≤ Twice a week, and 3 ≤ Three times a week), and the seven components form a global sleep quality score that ranges from 0 (good quality) to 21 (poor quality) (50). In this study, Cronbach's alpha was 0.801, and the value of 5 was used as the standard in selecting students with poor sleep quality.

### Statistical analysis

First, a factor analysis was used for testing common method bias. Secondly, the descriptive statistics, Chi square test, *t*-tests, and Pearson's correlation analysis were conducted using Stata version 15.0 (Stata Corporation, TX, USA). *N* (%) indicated data for categorical variables, mean ± SD indicated data for numerical variables. Pearson's correlations were used to test our first hypothesis and to examine the relationships among mobile phone addiction, sleep disturbances, and depression symptoms. The third step was to test the mediation model and moderated mediation effect model. Model 4 of the PROCESS macro (version 3.4) of the Statistical Package for the Social Sciences (SPSS) version 23.0 (51) was used to test the mediating effect of sleep disturbances. Model 7 of the PROCESS macro (version 3.4) was used to test the moderating role of gender on the mediation effect. In order to eliminate the units of measurement differences and make the relative strength of different variables comparable, we standardized all continuous variables in the model (52).

## Results

### Common method biases

To avoid common method biases, this study was carried out by collecting anonymous responses and scoring some items in reverse. And the common method biases was tested by Harman's single-factor test. Study findings showed that the KMO value is 0.896, the significance of Bartlett test is *P* < 0.001, and there were 11 factors with the original root >1. The first factor could explain 23.138% of the cumulative variance, and the critical value was <40%. It indicated that there is no serious common method bias in this study.

### Descriptive and correlational analyses

Before data analysis, the missing data were handled by mean imputation in Stata. Controlling for the effects of age, gender, residence, and self-rated health, partial correlation analysis was used. The descriptive statistics and correlation matrix are displayed in Table 2. Positive correlations were found between mobile phone addiction and depressive symptoms, the total score of sleep disturbances, sub-dimensions of sleep disturbances (subjective sleep quality, sleep latency, sleep duration, sleep disturbance, and daytime dysfunction). Depressive symptoms were positively correlated with the total score of sleep disturbances and seven dimensions of sleep disturbances. Age has significant positive correlations with mobile phone addiction and sleep disturbances, sub-dimensions of sleep disturbances (sleep disturbance, daytime dysfunction), but has no significant correlation with other variables. There were significant positive correlations between self-rated health and mobile phone addiction, depressive symptoms, the total score of sleep disturbances, sub-dimensions of sleep disturbances (subjective sleep quality, sleep latency, sleep disturbance, use of sleep medication, daytime dysfunction).

**Table 2:** Pearson's correlations among relevant study variables.

**Variables**	**1**	**2**	**3**	**4**	**5**	**6**	**7**	**8**	**9**	**10**	**11**	**12**	**13**	**14**
1. Age	1													
2. Gender	0.10	1												
3. Residence	0.00	−0.02	1											
4. Self-rated health	0.06	−0.09	0.03	1										
5. MPAI	0.16^***^	−0.03	0.06	0.17^***^	1									
6. PHQ9	0.08	0.05	−0.02	0.28^***^	0.45^***^	1								
7. PSQI	0.12^**^	0.04	0.02	0.27^***^	0.36^***^	0.57^***^	1							
8. Subjective sleep quality	0.04	−0.02	−0.02	0.27^***^	0.25^***^	0.41^***^	0.69^***^	1						
9. Sleep latency	0.08	0.07	−0.02	0.14^***^	0.18^***^	0.36^***^	0.68^***^	0.46^***^	1					
10. Sleep duration	0.00	−0.06	0.04	0.017	0.12^**^	0.12^**^	0.47^***^	0.18^***^	0.14^***^	1				
11. Habitual sleep efficiency	−0.02	0.01	0.02	0.00	0.02	0.12^**^	0.42^***^	0.11^**^	0.15^***^	0.27^***^	1			
12. Sleep disturbance	0.14^***^	0.04	−0.05	0.18^***^	0.24^***^	0.40^***^	0.58^***^	0.37^***^	0.31^***^	0.04	0.08	1		
13. Use of sleep medication	0.04	0.04	0.07	0.128^***^	−0.02	0.16^***^	0.2818^***^	0.10	0.12^**^	0.00	0.10	0.10	1	
14. Daytime dysfunction	0.19^***^	0.05	0.03	0.26^***^	0.43^***^	0.54^***^	0.73^***^	0.40^***^	0.33^***^	0.16^***^	0.11	0.41^***^	0.11^**^	1

* *p* < 0.05;

** *p* < 0.01;

*** *p* < 0.001.

MPAI, mobile phone addiction index; PHQ9, patient health questionnaire-9; PSQI, Pittsburgh Sleep Quality Index.

### Mediation effect analysis

Model 4 from the SPSS macro PROCESS 3.4 was used to test for the existence of mediation. In the absence of the mediator, mobile phone addiction was positively associated with depression symptoms after controlling for age, residence, and self-rated health. As shown in Table 3, mobile phone addiction significantly positively correlated with sleep disturbances. Both mobile phone addiction and sleep disturbances significantly positively correlated with depression symptoms. These results indicated that sleep disturbances partially mediated the relationship between mobile phone addiction, and depressive symptoms, which was consistent with hypothesis 1 and hypothesis 2.

**Table 3:** Testing the mediation model of sleep disturbances.

	**PSQI**				**PHQ9**			
			**BOOSTRAP 5000 TIMES 95% CI**				**BOOSTRAP 5000 TIMES 95% CI**	
	**β**	**S.E**.	**LLCI**	**ULCI**	**β**	**S.E**.	**LLCI**	**ULCI**
Constant	−2.31	1.6	−5.46	0.84	−6.86^***^	2.06	−10.89	−2.82
MPAI	0.07^***^	0.01	0.06	0.08	0.09^***^	0.01	0.07	0.11
PSQI	−	−	−	−	0.64^***^	0.04	0.556	0.73
Age	0.19^*^	0.08	0.03	0.34	0.169	0.1	−0.03	0.37
Residence	−0.06	0.17	−0.39	0.27	−0.39	0.22	−0.81	0.03
Self-rated health	0.77^***^	0.11	0.57	0.98	0.75^***^	0.14	0.48	1.02
	*R*^2^ =0.18				*R*^2^ = 0.41			
	F = 48.41^***^				F = 122.25^***^			

* *p* < 0.05;

** *p* < 0.01;

*** *p* < 0.001.

Variables have been normalized. 95% CI estimated using bootstrap method. Bootstrap sample size = 5,000.

CI, confidence interval; β, standardized regression coefficient; S.E., standard error; LLCI, lower level confidence interval; ULCI, upper level confidence interval; MPAI, mobile phone addiction index; PHQ9, patient health questionnaire-9; PSQI, Pittsburgh Sleep Quality Index.

**Figure 1:**
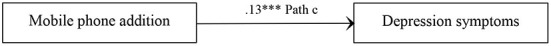
(Hypothesis 1) Total effect models: effect of mobile phone addiction on depression symptoms ***p < 0.001.

**Figure 2:**
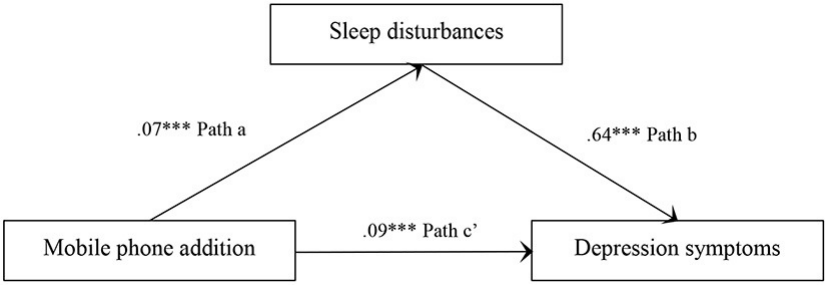
(Hypothesis 2) Mediation models: effect of mobile phone addition on depression symptoms with the mediation of sleep disturbances ***p < 0.001.

Figures 1, 2 show the total effect model and the mediation model. The results indicated that the three simple path coefficients (paths a, b, and c) were statistically significant. The results from 5,000 bootstrapping samples presented that all indirect effects were statistically significant, with the bootstrapping 95% CI excluding zero. The total effect of mobile phone addiction on depression symptoms was 0.13 (*p* < 0.001). The indirect effect of sleep disturbances was 0.04, 95% CI (0.03, 0.06), accounting for 33.09% of the total effect.

**Figure 3:**
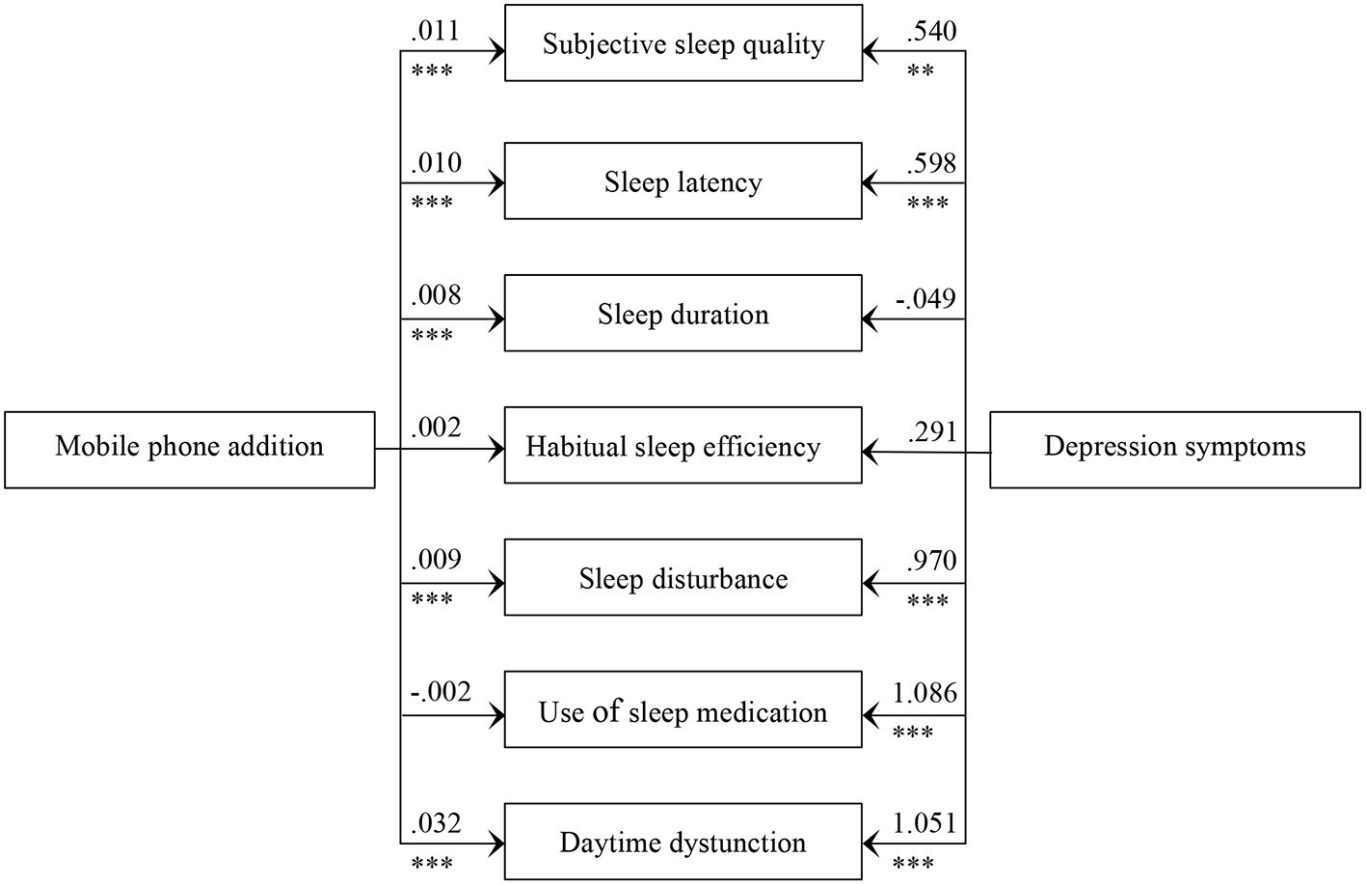
Mediating role of seven components of sleep disturbances on the relationship between mobile phone addiction and depression symptom ^*^p < 0.05; **p < 0.01; ***p < 0.001.

Subsequently, we further examined the parallel mediating effect of seven dimensions of sleep disturbances on the relationship between mobile phone addiction and depressive symptoms. As presented in Figure 3, there was a significant positive correlation between mobile phone addiction and five dimensions of sleep disturbances (subjective sleep quality, sleep latency, sleep duration, sleep disturbance, and daytime dysfunction). Four dimensions of sleep disturbances (subjective sleep quality, sleep latency, sleep disturbance, and daytime dysfunction) had a significant mediating effect on the relationship between mobile phone addiction and depressive symptoms, while other factors had no significant mediating effect. The indirect effect of subjective sleep quality was 0.01, 95% CI (0.00, 0.01); the indirect effect of sleep latency was 0.01, 95% CI (0.00, 0.01); the indirect effect of sleep disturbance was 0.01, 95% CI (0.00, 0.02); the indirect effect of daytime dysfunction was 0.03, 95% CI (0.02, 0.05).

### Moderated mediation effect analysis

**Figure 4:**
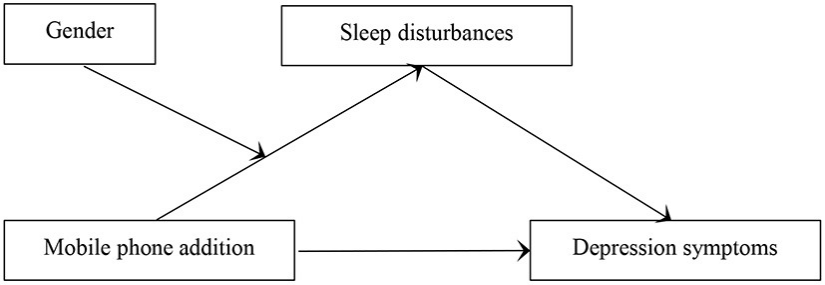
(Hypothesis 3) Moderated-mediation model: effect of mobile phone addiction on depression symptoms with sleep disturbances as a mediator and gender as a moderator.

Age, residence, and self-rated health were controlled, Model 7 from the SPSS macro PROCESS was applied to test for the proposed moderated mediation model with sleep disturbances as mediator and gender as a moderator. The relations between variables were illustrated in Figure 4. As shown in Table 4, mobile phone addiction positively correlated with sleep disturbances. Sleep disturbances and mobile phone addiction significantly positively correlated with depression symptoms. These results indicated that sleep disturbances partially mediated the relationship between mobile phone addiction and depression symptoms. However, the interaction between sleep disturbances and gender was not correlated with mobile phone addiction. These results indicated that gender could not moderate the relationship between mobile phone addiction and sleep disturbances, which is inconsistent with hypothesis 3 in our study.

**Table 4:** Testing the moderated mediation effect of mobile phone addiction on depression symptoms.

	**PSQI**				**PHQ9**			
			**BOOSTRAP 5000 TIMES 95% CI**				**BOOSTRAP 5000 TIMES 95% CI**	
	**β**	**S.E**.	**LLCI**	**ULCI**	**β**	**S.E**.	**LLCI**	**ULCI**
Constant	−0.036	1.59	−3.16	3.08	−3.62	2.04	−7.61	0.38
MPAI	0.090^***^	0.02	0.05	0.13	0.09^***^	0.01	0.07	0.11
PSQI	–	–	–	–	0.64^***^	0.04	0.56	0.73
Gender	0.342^*^	0.17	0.01	0.678	–	–	–	–
MPAI × Gender	−0.01	0.01	−0.04	0.01	–	–	–	–
Age	0.17^*^	0.08	0.02	0.33	0.17	0.10	−0.03	0.37
Residence	−0.05	0.17	−0.38	0.28	−0.39	0.22	−0.81	0.03
Self-rated health	0.76^***^	0.11	0.55	0.96	0.75^***^	0.14	0.48	1.02
			*R*^2^ = 0.43				*R*^2^ = 0.41	
			F = 33.27^***^				F = 122.25^***^	
	**Direct effect of MPAI on PHQ9**			
			**BOOSTRAP 5000 TIMES 95% CI**	
	β	**S.E**.	**LLCI**	**ULCI**
	0.09^***^	0.01	0.07	0.11
	**Conditional indirect effect at gender values**			
			**BOOSTRAP 5000 TIMES 95% CI**	
	β	**BootS.E**.	**BootLLCI**	**BootULCI**
M – 1 SD	0.05	0.01	0.04	0.06
M	−0.01	0.01	−0.03	0.01
M + 1 SD	0.04	0.01	0.03	0.05

* *p* < 0.05;

** *p* < 0.01;

*** *p* < 0.001.

Variables have been normalized. 95% CI estimated using bootstrap method. Bootstrap sample size = 5,000.

CI, confidence interval; β, standardized regression coefficient; S.E., standard error; LLCI, lower level confidence interval; ULCI, upper level confidence interval; MPAI, mobile phone addiction index; PHQ9, patient health questionnaire-9; PSQI, Pittsburgh Sleep Quality Index.

## Discussion

Although there have been many studies on the association between mobile phone addiction and depressive symptoms, the potential mechanisms underlying this process remain to be explored. Therefore, we proposed a moderated mediation model to examine the role of sleep disturbances and gender in this process. The results indicate that mobile phone addiction could significantly predict depressive symptoms. Besides, sleep disturbances played a mediating role in the relationship between mobile phone addiction and depressive symptoms, but the mediating role was not moderated by gender among Chinese University students.

Firstly, consistent with our hypothesis, the results indicate that mobile phone addiction could be a risk factor for depressive symptoms. Mobile phone addiction becomes more compulsive during the process of reinforcement, which could cause individuals to start experiencing negative emotions when they are not engaging in the behavior (53). Worry, anxiety, and other negative emotions are displayed when not using mobile phones. According to the study of dysfunctional mental processes, irrational beliefs (about the self) can be identified in the context of excessive mobile phone use. For example, low self-esteem occurs when you cannot reach your significant other on your mobile phone, which translates into frequent irrational or distorted cognitions (e.g., “I'm not worthy of love”) (54). Such feelings of inferiority, insecurity, and fluctuating self-esteem are important factors in depressive symptoms.

Secondly, our study found that sleep disturbances partially mediated the relation between mobile phone addiction and depressive symptoms. Mobile phone addiction involves cognitive and behavioral disorders (55). On one hand, our results are in line with previous findings, which suggest a link between mobile phone addiction and sleep disturbances. From a physiological point of view, the timing of melatonin onset at night is affected by screen use, as mobile phones emit large amounts of blue light, and controlling the secretion of melatonin is a key factor in regulating health and circadian rhythms (56, 57). Persistent sleep deprivation caused by mobile phone addiction may harm one's immune system, leaving them vulnerable to various diseases (58). Another possible explanation is the large-scale phenomenon of “emotional contagion” in the era of the digital Internet (59). Mobile phones are an important channel to access the social networking space, browsing social media sites before bedtime could potentially have an impact on their mood, leading to sleep disturbances. On the other hand, this study advanced our understanding of how mobile phone addiction could lead to depressive symptoms. In general, the more time people spend on mobile phones, the less interaction they have in real life, which may lead to feelings of loneliness and depression. In addition, prolonged and excessive use of social media may evoke social comparisons, which have been shown to lead to negative emotions and depression (60). Our results supported the “I-PACE” model, which states that the disorder caused by addictive behavior is the consequence of the interaction between an individual's core characteristics and several moderating and mediating variables, and these variables are dynamic (38).

Finally, our study found that gender does not play a moderating role in the relationship between mobile phone addiction and sleep disturbances. It is consistent with the findings of Salehan and Negahban that the moderating effect of gender on mobile phone use is inconclusive (43). One possible reason is that discussing gender differences in the context of social orientation can explain differences between males and females in cognition, behavior, and how they view their social roles. Under the guidance of social orientation, different genders will develop their own behavior patterns and social roles, thus producing different emotions (61). While previous studies have selected young people as samples, there are significant differences in education level, occupation, and marital status (16). Our research samples were University students from western China who share a common social role as “students” in the campus setting, with similar educational level and marital status. They also bear similar social expectations, so they show similarities in the relationship between mobile phone addiction and sleep disturbances. Another possible reason is as follows: previous studies have demonstrated that higher rates of Internet and computer addiction among males (44), while higher rates of mobile phone addiction among females (62). In China, for undergraduates, most universities have a system that requires the lights and power to be turned off regularly on weekday nights. In addition, students in the dormitories need to take care of their roommates' sleeping schedules. These reasons may limit male students from using laptops at night. Due to the advantages of convenience, high performance and accessibility, mobile phones can replace computers in many functions. For example, the latest generation of smartphones allow individuals to participate in a wide range of online activities, such as browsing social networks, watching videos, and playing online games. As an important terminal of mobile Internet, the functions of mobile phones are constantly being updated. Therefore, under the restrictions of the campus environment, the proportion of mobile phone addiction among males may increase, while the gap with the ratio of mobile phone addiction among females will narrow. So, the moderating effect of gender was not found between mobile phone addiction and sleep disturbances.

Our findings have both theoretical and practical implications for reducing mobile phone addiction, depressive symptoms, and sleep disturbances. Theoretically, our study has preliminarily clarified the cross-sectional correlations and direction of effects among mobile phone addiction, seven dimensions of sleep disturbances, and depressive symptoms, and explored the mediating effect of sleep disturbances between mobile phone addiction and depressive symptoms. These efforts offered the foundation for the next step in the study of the association mechanism. Our findings broaden the objects of the maladaptive coping style in sleep disturbances and depressive symptoms. From a practical perspective, our findings have implications for the prevention of depression. Depression symptoms worsen over time, so early intervention is needed. Interventions for sleep disturbances may be appropriate to alleviate mental health problems. There are three strategies to help correct mobile phone addiction: capacity-enhancing strategies (i.e., enhanced self-discipline and rational management ability), behavior reinforcement strategies (i.e., focus on real social networks that can interact face to face), information-enhancing strategies (i.e., support from friends can improve addictive behavior for those who fully understand the risks of mobile phone use) (63). Based on this, we propose the following suggestions for interventions.

First, technology-based interventions are necessary. Mobile software developers should further develop effective strategies to intervene in the link between mobile phone addiction and mental health problems. For example, they could create programs to limit the amount of time a mobile phone can be used. By providing the user with periodic reminders, warning signs that a running application is about to exit, or forced dormancy. Restrict mobile phone access during certain hours, such as at night or when users need to concentrate. To urge mobile phone addicts to better understand their phone usage habits, the application can regularly give users feedback on their daily phone usage, daily usage, duration, and unlock times of the phone. So that users can understand the daily duration of mobile phone use and the reasons and motivations behind mobile phone addiction. The direction of future software developers should be to provide personalized digital interventions based on the mobile phone usage habits of different users (64).

Second, University educators should publicize the potential hazards of mobile phone addiction, sleep disturbances, and depression symptoms. At the same time, University students are encouraged to sleep regularly, exercise moderately, control the use and dependence of mobile phones by improving self-control. University educators could present relevant coping strategies, such as encouraging students to set goals to reduce mobile phone use, sleep on time, and make efforts to stick to those goals. Through class meetings, lectures, psychological counseling, and other relevant practices, strengthen communication, timely relief of negative emotions. Meditation and self-control training may also be beneficial. One of the pathways leading to problematic mobile phone use is the extroversion pathway, where an individual's mobile phone addictive behavior is driven by a strong constant desire to communicate with others and establish new relationships (6). Therefore, we suggest that increasing communication and interaction with others in real life and finding catharsis ways can help improve mobile phone addiction behaviors.

Third, students could try to mobilize social support, such as mobilizing roommates to help each other, or establishing mutual aid groups in the class or dormitory. Classmates or dormitory members remind each other to exercise regularly and sleep on time, which may form a supervision mechanism among students and help them gradually develop good living habits. Guiding students to pour out troubles to families or friends is a good way of easing off pressure, reduce negative emotions, and improve sleep disturbances. For individuals with high social support, the association between problematic mobile phone use and negative emotions became insignificant (35).

There are some limitations to our research. First, in this study the measurements of MPAI, PSQI, and PHQ9 reported by participants, which naturally generates some reliability and validity questions, including the problem of recall bias and response style bias. Future studies can adopt multiple methods and include more information providers in the sample. The second limitation is that this was a University sample, which may add to the limitations of generalizability of the results to other samples. And we used measures of depressive symptoms rather than diagnostic assessments of depression, so any conclusions cannot be generalized to the clinical population. Further studies could examine these relationships using clinical populations. Thirdly, this was a cross-sectional design study, it could not detect causality due to the cross-sectional nature of the data. Longitudinal studies are expected in the future. Finally, our current study only considered depression symptoms, but sleep disturbances may mediate the association between mobile phone addiction and other psychological health concerns. Therefore, we recommend that future studies consider issues such as stress, loneliness, and anxiety. Despite these limitations, however, this study further examined the parallel mediating effect of seven dimensions of sleep disturbances on the relationship between mobile phone addiction and depressive symptoms. It also provides a new intervention strategy from the perspective of sleep disturbances to reduce depressive symptoms.

## Conclusion

Overall, mobile phone addiction is an important public health problem related to depressive symptoms and sleep disturbances. The result of the current study revealed that mobile phone addiction can be a risk factor for depressive symptoms of University students. Furthermore, this relationship could be mediated by sleep disturbances. Besides, gender did not moderate the indirect pathway between mobile phone addiction and depressive symptoms. Implications of the findings were discussed in the context of mobile phone addiction and psychological problems among University students. Identifying the key factors in the relationship between mobile phone addiction and depressive symptoms is important for more effective and well-targeted health interventions to prevent and treat depression across the life course. Our results also suggest the importance of early intervention among University students with mobile phone addiction, especially among those with sleep disturbances.

## Data availability statement

The original contributions presented in the study are included in the article/Supplementary material, further inquiries can be directed to the corresponding author.

## Ethics statement

The study protocol was reviewed and approved by Biomedical Ethics Committee of Xi'an Jiaotong University, China. Written informed consent for participation was not required for this study in accordance with the national legislation and the institutional requirements.

## Author contributions

CL designed the research, created the protocol, obtained ethical approval, contributed to the data analysis, and participated in revising the manuscript. ML provided support toward the design of the study, analyzed the data, and produced and revised the research manuscript. All authors read and approved the final manuscript.

## Conflict of interest

The authors declare that the research was conducted in the absence of any commercial or financial relationships that could be construed as a potential conflict of interest.

## Publisher's note

All claims expressed in this article are solely those of the authors and do not necessarily represent those of their affiliated organizations, or those of the publisher, the editors and the reviewers. Any product that may be evaluated in this article, or claim that may be made by its manufacturer, is not guaranteed or endorsed by the publisher.

## Abbreviations

SD: Standard Deviation
MPAI: Mobile Phone Addiction Index
PHQ9: Patient Health Questionnaire-9
PSQI: Pittsburgh Sleep Quality Index
CI: Confidence Interval
β: Standardized Regression Coefficient
S.E.: Standard Error
LLCI: Lower Level Confidence Interval
ULCI: Upper Level Confidence Interval.
